# Informatics Enhanced SNP Microarray Analysis of 30 Miscarriage Samples Compared to Routine Cytogenetics

**DOI:** 10.1371/journal.pone.0031282

**Published:** 2012-03-05

**Authors:** Ruth B. Lathi, Megan Loring, Jamie A. M. Massie, Zachary P. Demko, David Johnson, Styrmir Sigurjonsson, George Gemelos, Matthew Rabinowitz

**Affiliations:** 1 Stanford Fertility and Reproductive Medicine Center, Palo Alto, California, United States of America; 2 Natera, Redwood City, California, United States of America; University of Florence, Italy

## Abstract

**Purpose:**

The metaphase karyotype is often used as a diagnostic tool in the setting of early miscarriage; however this technique has several limitations. We evaluate a new technique for karyotyping that uses single nucleotide polymorphism microarrays (SNP). This technique was compared in a blinded, prospective fashion, to the traditional metaphase karyotype.

**Methods:**

Patients undergoing dilation and curettage for first trimester miscarriage between February and August 2010 were enrolled. Samples of chorionic villi were equally divided and sent for microarray testing in parallel with routine cytogenetic testing.

**Results:**

Thirty samples were analyzed, with only four discordant results. Discordant results occurred when the entire genome was duplicated or when a balanced rearrangement was present. Cytogenetic karyotyping took an average of 29 days while microarray-based karytoyping took an average of 12 days.

**Conclusions:**

Molecular karyotyping of POC after missed abortion using SNP microarray analysis allows for the ability to detect maternal cell contamination and provides rapid results with good concordance to standard cytogenetic analysis.

## Introduction

First trimester miscarriages are common among couples with up to 20% of clinically recognized pregnancies ending in spontaneous abortion [1]–[3]. These events are multifactorial; however, certain risk factors are known to increase the risk of miscarriage. These factors include older age, history of previous miscarriage, substance exposure, maternal extremes of weight, delayed ovulation to implantation interval, maternal systemic illness and uterine anomalies. Despite a multitude of maternal factors that can contribute to miscarriage, the majority (50–60%) of first trimester miscarriages are due to fetal chromosomal abnormalities [4].

Whereas chromosomal testing of products of conception (POC) is not recommended for every miscarriage, there are many scenarios where knowing the chromosome status of a miscarried fetus can help in clinical management. It can be particularly helpful in the recurrent pregnancy loss and infertility populations. The most common method of testing is a metaphase karyotype, which is available through the cytogenetics department in most hospitals [5]. This method is considered the gold standard for chromosome analysis but has three practical limitations. First, a successful cell culture is required but failure occurs in 10–40% of cases (6). Second, the results take approximately 4–6 weeks. And third, if the results suggest normal female karyotype (46,XX), a result that happens 55–80% of the time, it is unknown whether the tested sample was of fetal or maternal in origin [6].

We evaluate here, in a blinded, head-to-head fashion, a new informatics enhanced technique that uses genotypic data of both the POC sample and the mother, measured with single nucleotide polymorphism (SNP) microarrays, to detect the number of copies of all 24 chromosomes simultaneously [7]. These arrays afford faster turnaround time and when combined with the Parental Support™ algorithm are able to determine parental source of chromosomes and abnormalities. Minimal tissue is required for karyotype analysis using SNP microarrays and the technology is able to confidently differentiate between maternal and fetal chromosomes in case of 46,XX.

The objective of this study is to examine the efficiency and accuracy of the informatics based technique in combination with single nucleotide polymorphism microarrays on products of conception after first trimester miscarriage in a prospective cohort.

## Materials and Methods

This study was approved by the Stanford University Institutional Review Board and all subjects gave written and verbal consent to participate. Couples treated at an academic reproductive endocrinology and infertility practice with a documented intrauterine pregnancy loss between February 2010 and August 2010 were eligible. Patients were offered enrollment if both parents were available to give DNA samples and desired chromosome testing of the miscarried tissue.

A missed abortion was diagnosed by transvaginal ultrasound and confirmed by repeat ultrasound prior to the dilation and curettage (D&C) procedure [8]. Suction curettage was performed in usual fashion under ultrasound guidance. Chorionic villi were separated from maternal deciduas via a standardized technique [9]. Once chorionic villi were separated and cleaned, the specimen was divided into equal samples and sent for microarray testing in parallel with routine cytogenetic testing.

Thirty samples were analyzed by both microarray and traditional cytogenetics. The mean age of women was 37.2 years old; range 29–41 years. Mean maternal body mass index (BMI) was 26.7 kg/m^2^; range 20–32. Table 1 includes all demographic data from the study population. The mean gestational age at time of D&C was 8.75 weeks; with a range 7–12 weeks; 27% of pregnancies were spontaneous conceptions whereas 73% were conceived using assisted reproductive technologies (33% intrauterine insemination (IUI), 37% in vitro fertilization (IVF), and 3% IVF with donor egg).

**Table 1:** Demographic data.^a^

Maternal Age (yr)	37.2 (29–41)
Paternal Age	37.9 (30–49)
Maternal BMI	26.7 (20–32)
Mode of conception	
Spontaneous	27%
IUI	33%
IVF	37%
Donor oocyte	3%
Gestational age at time of D&C (wks)	8.75 (7–12)
Maximum CRL achieved (mm)^b^	12 (6–29)
Prior live births	
0	67%
1	30%
2	3%
Prior miscarriages	
0	46%
1	20%
2	17%
3 or more	17%

a Results expressed as mean and range, with exceptions noted.

b In 10 of the 30 cases, no fetal pole was present, with only a gestational sac visualized on ultrasound.

Genotyping of the maternal and POC samples was performed at a commercial reference lab using Illumina CytoSNP-12 genotyping microarrays, which measure approximately 300,000 SNPs across the genome, (roughly one every 10 kb) according to the manufacturer's instructions. After a genomic sample is run on a SNP array the results must pass a rigorous in-house quality control test before further analysis is done. The informatics technique (Parental Support™), using the output of the SNP arrays, determined the number and origin of each of the chromosomes in the POC sample.

**Figure 1:**
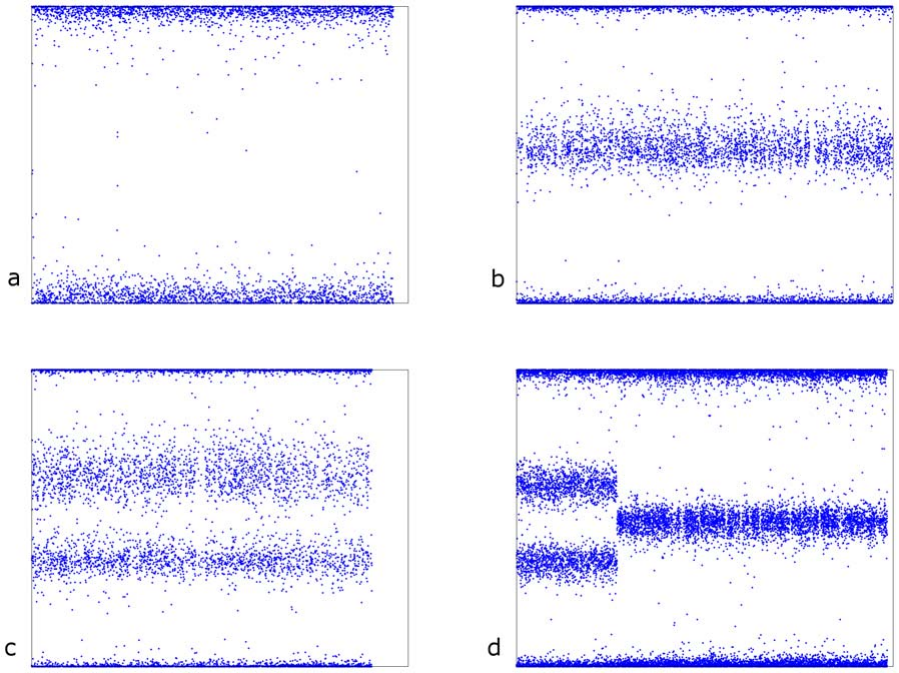
Graphic illustration of data for individual chromosome with different chromosome copy number a) monosomy 21, b) disomy 6, c) trisomy 16, d) partitial duplication chromosome 7.

In order to determine the chromosome copy number, the informatics technique compares, for each SNP on a given chromosome, the intensity of the signal from each of two possible alleles. The relative intensities have a statistical distribution that is indicative of the number of copies of that chromosome. Each analyzed sample produces 24 images, one for each chromosome, and the chromosome number is determined for each chromosome based on the image produced. Whereas the majority of chromosomes will be disomic as in Figure 1b, abnormalities such as monosomy, trisomy, segmental additions or deletions will appear clearly different from disomic chromosomes. Triploidy is detected when all chromosomes are trisomic. The images in Figure 1 are for individual chromosomes graphically illustrating different ploidy states; each plot represents one chromosome, and each dot represents one SNP. The x-axis is the SNP location along the chromosome, and the y-axis is the relative intensity of the two allelic measurements at that SNP. SNPs that are homozygous are found either at the top or the bottom of the plot, While snps that are heterozygous are toward the center of the plot.

In the case of a monosomy (Fig. 1a), alleles are all homozygous so we only see the top and bottom cluster. In the case of a disomy (Fig. 1b), we see the two sets of homozygous alleles at the top and bottom, and also the set of heterozygous alleles in the middle. In the case of a trisomy (Fig. 1c), we see the two sets of homozygous alleles plus two sets of heterozygous alleles (AAB and ABB). Figure 1d shows an example of a partial duplication.

Routine cytogenetic testing consisted of cell culture and analysis by the Giemsa-trypsin-wrights banding methods by a university reference lab. Chorionic villi from a miscarriage were grown in culture, the cells were then arrested and Giemsa used to stain bands in the chromosomes to allow for visualization and identification, known as G-banding.

## Results

Overall, 63% of karyotyped POC were chromosomally abnormal on routine cytogenetics. The mean maternal age at the time of miscarriage was 37.2 years and the mean gestational age at the time of uterine evacuation was 8.75 weeks (see Table 1 for patient characteristics). The majority of abnormalities were due to autosomal trisomies (15 out of 19) and, not surprisingly, 14 of 15 of these were due to excess maternal chromosomes. In addition to the autosomal trisomies, there were two unbalanced translocations (one inherited and one de novo). There was one triploidy and one tetraploidy in our data set. All 46,XX results from cytogentics were confirmed to be POC samples as microarray analysis clearly detected one maternal and one paternal copy of each chromosome, ruling out maternal contamination. The cytogenetic karyotyping took, on average, 29 days to return results. In contrast, the microarray based karyotyping took 12 days for the initial research samples. The last eight microarray samples, run in a production setting, averaged only 7 days for turn around.

The chromosome results of the 30 samples from each of the 2 techniques are shown in Table 2. Of the 30 samples, four results were discordant between the cytogenetic and microarray techniques, giving a concordance of 87% between the two techniques. In one case, cytogenetic karyotyping returned a 92,XXXX result, while the informatics technique found 46,XX. In a second case, cytogenetics reported a 46,XY,+15,der(15;15)(q10;q10) karyotype, while the informatics technique returned a result of trisomy 15. In a third case, cytogenetics found trisomy 22, while the informatics technique found maternal cell contamination (testing separate samples from the same case). In the last case, the cytogenetics reported a 46, X, +22 karyotype, while the informatics technique reported 47,XX, +22.

**Table 2:** Karyotype results of 30 cases analyzed by both SNP micro arrays and cytogenetics.

Abnormalities Detected	SNP micro array	cytogenetics
Single Autosomal Trisomies*	14	14
Double trisomy	2	2
Triploidy 69,XXY	1	1
Tetraploidy 92,XXXX	0	1
Unbalanced translocation**	1	2
MCC	1	0
Normal 46,XX non-maternal	9	8
Normal 46,XY	2	2

* Autosomal Trisomies identified (2,3,13,14,15,16,18,20,22).

** One unbalanced translocation detected by both methods 46,XX, der (14)t(3;14)(p21;q32)mat.

One unbalanced robertsonian translocation 46,XY,+15,der(15;15)(q10;q10) was reported as trisomy 15 by SNP micro array.

The first two cases with a discordant result can be easily explained by the known limitation of quantitative genetics and their inability to detect whole genome duplication and balanced structural rearrangements. However, only one would be considered a false negative result (meaning that the reported result was normal non-maternal when the true karyotype of the POC was tetraploid). The missed unbalanced structural rearrangement was reported as abnormal and lethal. In this case the parental karyotypes were normal and the translocation was considered denovo, and no more likely to recur than a denovo aneuploidy. The latter two cases of discrepancies illustrate a limitation of the study. Although the samples analyzed by the two methods came from the same procedure, the tissue sample analyzed was different and, therefore, the discrepancies can be explained by sampling error, tissue mosaicism, or culture artifact. Again, in the case or trisomy 22 with or without monosomy X, the patient would be counseled the same and no further evaluation for maternal causes of miscarriage are indicated.

## Discussion

The application of informatics and microarray technology to analysis of miscarriage tissues gives fetal karyotype results in significantly less time than routine cytogenetic testing, and avoids the potential pitfalls of cell culture. While array comparative genomic hybridization (aCGH) has been reported in this context [10], [11], to our knowledge, this is the first application of SNP microarray technology to determining the karyotype of POC samples. The use of the informatics enhanced SNP microarray technique gives us additional information about parental source of aneuploidy, enables detection of uniparental disomy (UPD), and is able to differentiate POC from maternal DNA in the setting of a 46,XX result.

Although definitive testing is not routinely done in the case of a 46,XX result, studies show that maternal contamination is present in 29–58% of cases where 46,XX is reported with routine cytogenetics [6]. In a study by Robberecht et al, 105 miscarriages were studied with both conventional cytogenetics and a CGH. Although they were able to identify 7 cases of likely MCC due to discrepancies between cytogenetic and aCGH, there were still a significant excess of 46,XX cases detected by both methods (37 cases of 46,XX compared to 4 cases of 46,XY) [12]. The excess was attributed to maternal contamination, though not confirmed with any molecular markers. The inability to trust a 46,XX result leads the patient and provider to either disregard the information given or conclude the miscarriage was chromosomally normal when this may or may not be an accurate reflection of the miscarriage. Being able to differentiate maternal contamination from a chromosomally normal female improves the accuracy of this test significantly.

The informatics enhanced microarray technology has two limitations compared to cytogenetic karyotyping: (a) it cannot detect errors due to duplication of the entire genome, and (b) it cannot detect balanced structural rearrangements. On the other hand, the informatics enhanced microarray method described herein can detect errors where the identities of chromosomes are wrong, e.g. UPD, a condition that other technologies such as fluorescence in situ hybridization (FISH), aCGH, and cytogenetic karyotyping cannot detect. Two of the discordant results illustrate the two limitations of the microarray informatics based technique: (a) it did not detect a 2∶2 tetraploidy; and (b) nor did it detect a Robertsonian translocation.

Tetraploidy is found in 2% of POC samples [4] and can be classified into two categories: the more common type, 2∶2 tetraploidy, is where each parent contributes a full extra set of chromosomes for a total of 2 identical sets from the mother and 2 identical sets from the father, and is likely due to failure of cytokinesis at all of the chromosomes. The less common, 3∶1 tetraploidy, is where 3 sets of chromosomes are from one parent and 1 is from the other, and has a more complicated origin. SNP microarrays are able to detect triploidy (3 chromosome sets) due to their genotyping ability, and when using the informatics technique used in this study, able to detect 3∶1 tetraploidy. However detection of multiples of entire chromosome sets, such as 2∶2 tetraploidy or 3∶3 hexaploidy, remains a limitation of the technology. These types of abnormalities can be detected with traditional cytogenetic karyotyping, or with microarray techniques used in combination with flow cytometry, or FISH [12]–[14].

Errors involving structural chromosome rearrangement, such as balanced translocations and Robertsonian translocations, are found in approximately 0.5% of POC samples [15]. Note that a Robertsonian translocation is formally a balanced translocation, although in practice, the complementary chromosome made up of the two tiny p-arms found in acrocentric chromosomes is often lost after a few rounds of mitosis. The inability to detect balanced chromosomal structural rearrangements, or differentiate between actual trisomy due to three separate copies of the chromosome versus a specious trisomy resulting from an isochromosome or Robertsonian translocation, is a limitation inherent to all microarray testing [16]. These types of abnormalities can only be detected with traditional cytogenetic techniques where the chromosome itself and banding pattern can be viewed. A balanced translocation is not lethal and, therefore, would not be expected to cause a miscarriage. If the genetic material is not balanced, then the array would be able to identify any unbalanced karyotype. Couples at risk for having balanced structural rearrangements in either parent should undergo routing cytogentic analysis of peripheral blood to accurately identify whether a translocation is present.

These two limitations are potentially clinically relevant since they can result in misdiagnosis of ploidy state in the event of 2∶2 tetraploidy, or, erroneous recurrence risk for subsequent miscarriage if a trisomy occurred due to an inherited unbalanced Robertsonian translocation when the presumption was pure trisomy. However, options exist for addressing these limitations. Trisomic results involving the acrocentric chromosomes (13, 14, 15, 21, 22) can be followed up with parental karyotyping. If a trisomy of an acrocentric chromosome is found with a microarray, the possibility or an isochromosome or balanced Robertsonian translocation in a parent exists. Therefore, providers are advised to proceed with parental karyotype if there is a clinical indication, such as infertility or recurrent pregnancy loss, because it has the potential to impact patient care and prognosis. An advantage of the informatics technique is the ability to determine the parental source of aneuploidy, enabling karyotyping to be done only on the parent of origin. To address tetraploidy, reflex testing of normal results with two-probe FISH would enable confirmation or detection of tetrasomic state leading to a conclusion of tetraploidy.

The other two discordant results are likely due to discordant samples. The case of maternal cell contamination is surprising only in that of sixty biopsies (samples from 30 study subjects ×2); only one was contaminated with maternal cells. Note: this includes eight cases of 46,XX as called by cytogenetic karyotyping, which were confirmed as genuine 46,XX by the microarray informatics technique. The final discordance was due to a finding of 46,X,+22 by cytogenetics, and a finding of 47,XX,+22 by the informatics technique. This may be due to a culturing artifact that resulted in a dropped chromosome in the cytogenetic sample, or to mosaicism in the POC sample, rather than testing error since the microarray informatics result delivered exceptionally clean data illustrating two distinct genotypes [see Fig. 1b]. The finding of discrepant results involving complex aneuploidies between cytogenetic and molecular methods (aCGH) have been previously reported and may be as high as 50% [15]. Here the authors postulate an alternative explanation of placental mosaicism that is revealed through analysis of direct versus cultured chorionic villi [17].

Though interesting, these discrepancies are not clinically significant in terms of misdiagnosis or incorrect prognosis. A 46,X,+22 and a 47,XX,+22 are both aneuploid and associated with the same clinical conclusion, and a finding of maternal contamination is simply interpreted as non-fetal.

Since the two types of aneuploidy that the informatics based technique can not detect typically account for approximately 3% of POC cases, this indicates that this technique should, in theory, be roughly concordant with 97% of the results from cytogenetic karyotyping, making the strong assumption of no cell culture artifacts or other errors during cytogenetic karyotyping. In this study, the concordance rate was 87% (26/30), with the clinically relevant concordance rate being 93% (28/30). The clinically relevant concordance was defined as differences in molecular karyotype and routine karyotype that would change how patients were counseled about the cause of their miscarriage. Given the small sample size of this study, we would expect the concordance to be higher with larger numbers as tetraploidy and Robersonian translocations are rare but both occurred in our sample.

All diagnostic tests have their advantages and disadvantages. Therefore, physicians must weigh the pros and cons of different testing options to counsel patients on their test options and results. Maternal cell contamination is a significant problem in POC diagnosis, and can often lead to spurious conclusions. The use of the careful tissue separation techniques, as previously described, can bring down the rate of MCC in POC samples dramatically, but maternal cell contamination can still occur [9]. Use of the SNP arrays combined with an informatics based technique such as the one described herein can identify fetal and maternal tissue, removing the uncertainty associated with MCC. Rates of culture failure and maternal contamination likely vary at different centers. If a physician encounters high culture failure rates (>5%) or disproportionately high numbers of 46,XX results with routine cytogenetics, he or she may consider molecular karyotyping with SNP arrays as a first line. However, there are some relatively rare abnormalities that can be missed, such as 2∶2 tetraploidy and robertsonian translocations and physicians using SNP microarrays should be aware of these.

Patients go though significant grieving after a miscarriage and knowing the results of the chromosomal analysis sooner may help patients understand the cause of their loss and begin planning for future pregnancy attempts. The informatics based technique described herein can bring the turn-around time of karyotyping from one month to less than a week. The benefits of rapid turnaround, the ability to definitively detect maternal contamination, the elimination of cell culture and its associated problems, justify the continued exploration and clinical application of this technology for analyzing products of conception.
