# Correction: Informatics Enhanced SNP Microarray Analysis of 30 Miscarriage Samples Compared to Routine Cytogenetics

**DOI:** 10.1371/annotation/7a3d2279-0f96-433c-bb3f-d7fda1759633

**Published:** 2012-08-10

**Authors:** Ruth B. Lathi, Jamie A. M. Massie, Megan Loring, Zachary P. Demko, David Johnson, Styrmir Sigurjonsson, George Gemelos, Matthew Rabinowitz

There was an error in the published author order. The correct by line is:

Ruth B. Lathi, Jamie A. M. Massie, Megan Loring, Zachary P. Demko, David Johnson, Styrmir Sigurjonsson, George Gemelos, Matthew Rabinowitz.

The correct citation is:

Lathi RB, Massie JAM, Loring M, Demko ZP, Johnson D, et al. (2012) Informatics Enhanced SNP Microarray Analysis of 30 Miscarriage Samples Compared to Routine Cytogenetics. PLoS ONE 7(3): e31282. 

